# LincRNA-p21 Levels Relates to Survival and Post-Operative Radiotherapy Benefit in Rectal Cancer Patients

**DOI:** 10.3390/life10090172

**Published:** 2020-08-31

**Authors:** Yan Li, Joan J. Castellano, Isabel Moreno, Francisco Martínez-Rodenas, Raquel Hernandez, Jordi Canals, Tania Diaz, Bing Han, Carmen Muñoz, Albert Biete, Mariano Monzo, Alfons Navarro

**Affiliations:** 1 Molecular Oncology and Embryology Laboratory, Human Anatomy Unit, Faculty of Medicine and Health Sciences, University of Barcelona, IDIBAPS, 08036 Barcelona, Spain; yanli@clinic.cat (Y.L.); joan.castellano@ub.edu (J.J.C.); fmartinez@bsa.cat (F.M.-R.); rahernandez@ub.edu (R.H.); canals.serrat@ub.edu (J.C.); tdiaz@ub.edu (T.D.); bhanhanx7@ub.edu (B.H.); carmen.munoz@ub.edu (C.M.); mmonzo@ub.edu (M.M.); 2 Department of Medical Oncology and Surgery, Hospital Municipal de Badalona, 08911 Badalona, Spain; imoreno@bsa.cat; 3 Radiation Oncology Department, Hospital Clinic de Barcelona, University of Barcelona, 08036 Barcelona, Spain; abiete@clinic.cat

**Keywords:** lincRNA-p21, colorectal cancer, radiochemotherapy, rectal cancer, long non-coding RNA, liquid biopsy

## Abstract

LincRNA-p21 is a long non-coding RNA involved in the p53 pathway and angiogenesis regulation that acts as prognostic marker in several tumors. In the present study, we aimed to analyze the clinical value of lincRNA-p21 in 177 resected stage I–III colorectal cancer (CRC) patients. Tumor and normal paired tissue and plasma samples from tumor-draining mesenteric veins and paired peripheral veins were analyzed. LincRNA-p21 expression was determined by RTqPCR and correlated with disease-free (DFS) and overall survival (OS). LincRNA-p21 was downregulated in tumor versus normal tissue (*p* = 0.0012). CRC patients with high lincRNA-p21 expression had shorter DFS (*p* = 0.0372) and shorter OS (*p* = 0.0465). Of note, the major prognostic impact was observed in the subset of rectal cancer patients where patients with high lincRNA-p21 levels had worse DFS (*p* = 0.0226) and OS (*p* = 0.0457). Interestingly, rectal cancer patients with high lincRNA-p21 benefited from post-operative chemoradiotherapy, as indicated by a longer OS in the group of high lincRNA-p21 patients receiving post-operative chemoradiotherapy (*p* = 0.04). Finally, patients with high lincRNA-p21 levels in mesenteric vein (MV) had shorter OS (*p* = 0.0329). LincRNA-p21 is a marker of advanced disease and worse outcome in CRC. Moreover, rectal cancer patients with high lincRNA-p21 levels could benefit from post-operative chemoradiotherapy, and plasmatic-lincRNA-p21 is a promising liquid biopsy biomarker.

## 1. Introduction

Colorectal cancer (CRC) remains the third most common cancer worldwide, with 1.8 million new cases per year (10.2%), and it is the second leading cause of cancer-related death, with almost 900,000 deaths annually (9.2%) [1]. CRC comprises two different cancer types—colon cancer (CC) and rectal cancer (RC)—which have traditionally been grouped together, although they are different diseases with a distinct prognosis and clinical management [2]. The treatment of early-stage CC is mostly based on surgery, with adjuvant chemotherapy in patients with lymph node involvement [3]. In contrast, although standard treatment of early-stage RC consists of surgery, if there are poor prognostic signs, then post-operative chemotherapy/chemoradiotherapy (CT/CRT) treatment could be included [4]. In more advanced stages, preoperative CRT followed by surgery is recommended [4]. Despite advances in the treatment of CRC, biomarkers to identify patients at high risk of relapse are needed to guide treatment options and improve survival rates [5], and non-coding RNAs are promising candidates [6].

Non-coding RNAs comprise 97% of the transcriptome, while protein-coding mRNAs account for only 3% [7]. Long non-coding RNAs (lncRNAs)—RNA molecules larger than 200 nucleotides lacking protein-coding potential [8]—have been related to the main hallmarks of cancer [9] and have been described as key mediators of tumorigenesis in different cancer types, including CRC [10]. One of the properties that make lncRNAs especially interesting as potential biomarkers is their highly specific expression pattern across tissues and diseases [11,12]. LncRNA expression has been shown to discriminate between tumor and normal tissue better than protein-coding genes [13]. In this setting, lncRNAs have gained interest as novel biomarkers for CRC determined in both tumor tissue and liquid biopsy samples [14,15].

The long intergenic non-coding RNA p21 (lincRNA-p21) was first described in 2010 as a *TP53*-activated regulator of p53-mediated apoptosis [16]. In recent years, lincRNA-p21 has been mainly linked to apoptosis regulation [17,18] and response to hypoxia [19,20]. However, other functions have also been reported, including the regulation of epithelial-to-mesenchymal transition [6]. However, the role of lincRNA-p21 in CRC remains poorly understood and explored only in vitro or using small cohorts of patients [21,22].

In the present study, we have explored the potential use of lincRNA-p21 expression levels in tumor tissue from resected CRC patients as a prognostic biomarker. We have assessed the expression levels of lincRNA-p21 in tumor and adjacent normal tissue exploring its association with overall survival (OS) and disease-free survival (DFS) in 177 CRC. We additionally performed an exploratory study to evaluate the role as a liquid biopsy biomarker of lincRNA-p21 using plasma samples obtained from the tumor-draining mesenteric vein (MV) or paired peripheral vein (PV).

## 2. Materials and Methods

### 2.1. Patient Samples

Tumor and paired adjacent normal tissue samples were obtained from 177 adult CRC patients at the time of surgical resection in the Hospital Municipal de Badalona, and all samples were stored at −80 °C until use. In addition, on the day of surgery, 5 mL of blood was drawn from the PV and stored in heparinized tubes. During surgery, with vascular ligation before tumor resection, an additional 5 mL of blood was drawn from the inferior tumor-draining mesenteric vein (MV) in 20 rectal cancer patients. Blood samples were collected in BD Vacutainer EDTA tubes (k3) and plasma were separated by centrifugation for 10 min at 2000× *g* and stored at −20 °C until use. The study was approved by the hospital Ethics Committee, and all participants provided their signed informed consent in accordance with the Declaration of Helsinki.

### 2.2. RNA Extraction from Tissue

RNA was isolated from tumor and normal tissue using TriZol™ Reagent (Life Technologies, Grand Island, NY, USA) and from plasma with TriZol™ LS Reagent (Life Technologies) supplemented with MS2 RNA using the Total RNA purification kit (Norgen, Thorold, ON, Canada) according to the manufacturer’s instructions. cDNA was obtained from 500 ng of RNA using the High-Capacity cDNA Reverse Transcription Kit (Applied Biosystems, Foster City, CA, USA). Relative lincRNA-p21 expression was determined by real-time polymerase chain reaction (RTqPCR) using the StepOne™ Real-time PCR System Thermal Cycling Block (Applied Biosystems). Quantification of lincRNA-p21 expression levels was performed with Custom qPCR Probes (Integrated DNA Technologies, Coralville, IW, USA) using the primers and probes described by Hall et al. [17]. Taqman gene expression assays of B2M (beta-2-microglobulin) (Hs99999907_m1) and 18 s (Hs99999901_s1) (Applied Biosystems) were used as endogenous control for tissue and blood samples, respectively.

### 2.3. Statistical Analyses

DFS was defined as the time between surgical resection and recurrence, death without recurrence, or last follow-up. OS was calculated from resection to the date of death or last follow-up. Kaplan–Meier curves for DFS and OS were plotted and compared with log-rank test. All factors with *p* < 0.1 in the univariate analysis were included in the Cox multivariate regression analyses of DFS and OS to determine hazard ratios (HR) with their 95% confidence intervals (CI). Paired (when necessary) or non-paired t-tests were used for comparisons between groups. Optimal cutoffs of lincRNA-p21 expression data for DFS and OS were obtained using X-Tile software [23]. All statistical analyses were performed using PASW Statistics 18 (SPSS Inc., IBM, Chicago, IL, USA), R 3.5.2 and GraphPad prism v8. 5.

## 3. Results

### 3.1. Patients

From 2003 to 2017, 177 surgically resected CRC patients not having received any treatment prior to surgery were included in the study. Neoadjuvant treatment was the unique exclusion criteria used in the patient selection, since it could affect lincRNA-p21 basal levels. Ninety-six patients had CC and eighty-one had RC. All patients had tumor and adjacent normal tissue samples and 20 RC patients had blood samples from the MV obtained during surgery and paired PV. Ninety-two (52%) received adjuvant treatment (0 stage I, 42 stage II, and 50 stage III). Among the patients with CC, 51 received adjuvant CT (22 Capecitabine + Oxaliplatin; 17 Capecitabine; 10 Mayo scheme; 2 Folfox), while among those with RC, eight received adjuvant CT (5 Capecitabine; 1 Capecitabine + Oxaliplatin; 1 Mayo scheme; 1 Raltitrexed + Oxaliplatin) and 33 received CRT (19 Capecitabine plus radiotherapy [45–50.4 Gy] and 14 Capecitabine + Oxaliplatin plus radiotherapy [45–50.4 Gy]). Median follow-up time for CRC patients was 97.6 months (Interquartile range, IQR 65.3–161.7). During follow-up, 33 CRC patients relapsed, 22 of whom died. Table 1 displays the main clinical characteristics of the patients for the entire cohort and split in CC or RC alone.

### 3.2. LincRNA-p21 Expression Levels

**Figure 1:**
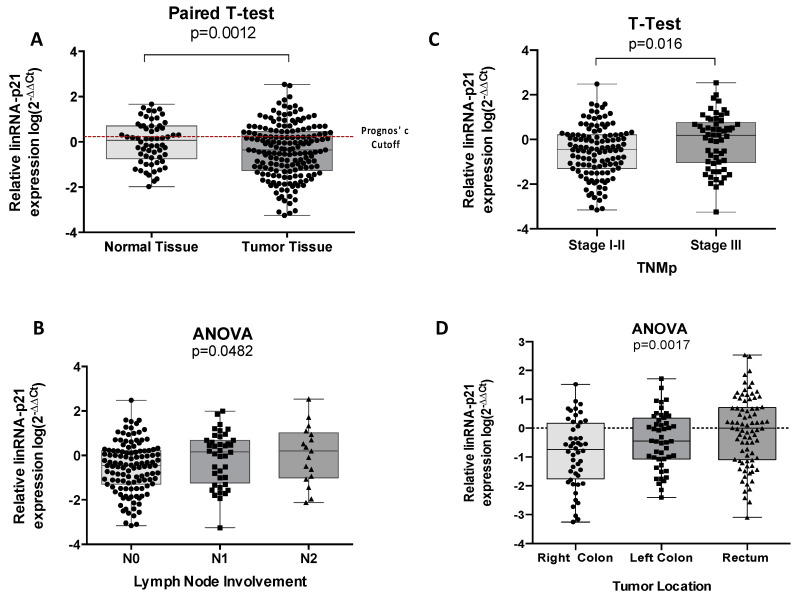
(**A**) Long intergenic non-coding RNA p21 (LincRNA-p21) expression in normal vs. tumor tissue. The prognostic cutoff used to divide patients in high or low expression for the prognosis analysis is indicated in the graph (red dashed line). (**B**) LincRNA-p21 expression according to lymph node involvement; (**C**) LincRNA-p21 expression in stage I–II vs. stage III disease; and (**D**) right/transverse colon vs. left colon vs. rectum.

First, we analyzed whether differences in the lincRNA-p21 levels exists between tumor and normal paired tissue. In this analysis, we only included a representative and randomly selected part of the normal tissues available (*n* = 62). LincRNA-p21 was downregulated in tumor compared to normal tissue samples (Paired t-test *p* = 0.0012) (Figure 1A). The correlation of lincRNA-p21 levels in tumor tissue with the main clinicopathological characteristics showed a significant association with lymph node involvement (N), disease stage, and tumor location. N+ patients had higher levels of lincRNA-p21 than N− patients (*p* = 0.016). Moreover, lincRNA-p21 levels were significantly different among N0, N1, and N2 patients (*p* = 0.0482) (Figure 1B). These results are in line with the association observed with disease stage where tumor samples from patients with stage III CRC (N+ patients) had higher levels of lincRNA-p21 than those with stage I–II (N− patients) disease (*p* = 0.016) (Figure 1C). Finally, we observed different licRNA-p21 levels according to tumor location, with increasing levels from right colon to rectum (*p* = 0.002) (Figure 1D).

### 3.3. LincRNA-p21 Expression and Survival

**Figure 2:**
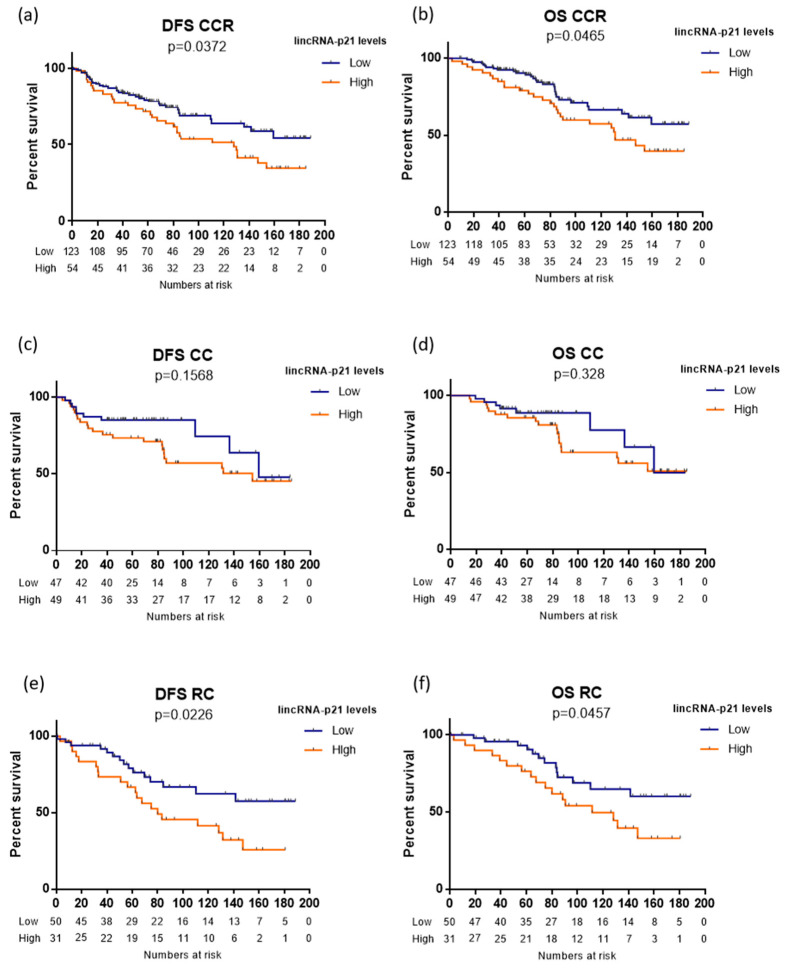
Disease-free survival (DFS) and overall survival (OS) according to lincRNA-p21 expression levels in (**a**,**b**) 177 colorectal cancer patients, (**c**,**d**) 96 colon cancer patients, and (**e**,**f**) 81 rectal cancer patients. The log-rank test was used to calculate whether significant differences in survival times between high or low lincRNA-p21 levels were achieved.

Using the optimal cutoff values identified by X-Tile, we classified the patients as having high or low lincRNA-p21 expression (Figure 1A). Of the 177 CRC patients, 135 had low and 42 had high expression. CRC patients with high lincRNA-p21 expression had shorter DFS (not reached [NR] versus 128.9 months; *p* = 0.0372; Figure 2a) and shorter OS (NR versus 131.5 months; *p* = 0.0465; Figure 2b) than those with low levels.

When we performed a sub analysis according to tumor location (CC versus RC), we observed that of the 96 CC patients, 47 had low and 49 high expression; and of the 81 RC patients, 50 had low and 31 had high expression. In the subgroup of 96 CC patients, despite the CC patients with high lincRNA-p21 levels having worse outcome, there was no significant association between lncRNA-p21 levels and either DFS or OS (Figure 2c,d). Interestingly, in the subgroup of 81 rectal cancer patients, those with high lincRNA-p21 levels had significantly shorter DFS (NR versus 80.1 months; *p* = 0.0226; Figure 2e) and OS (NR versus 111.6 months; *p* = 0.0457; Figure 2f).

### 3.4. LincRNA-p21 is an Independent Prognosis Marker in CRC

The multivariate analysis of DFS identified lincRNA-p21 as an independent prognostic marker in the overall cohort (HR 1.74, 95% CI 1.07–2.84; *p* = 0.025) and in the subgroup of rectal cancer patients (HR 1.98, 95% CI 1.01–3.8; *p* = 0.046). The multivariate analysis of OS identified lincRNA-p21 levels as an independent prognostic marker in all patients (HR 1.88 95% CI 1.10–3.21; *p* = 0.020). Table 2 shows all factors identified as independent markers in the multivariate analyses.

### 3.5. LincRNA-p21 and Chemoradiotherapy in Rectal Cancer

**Figure 3:**
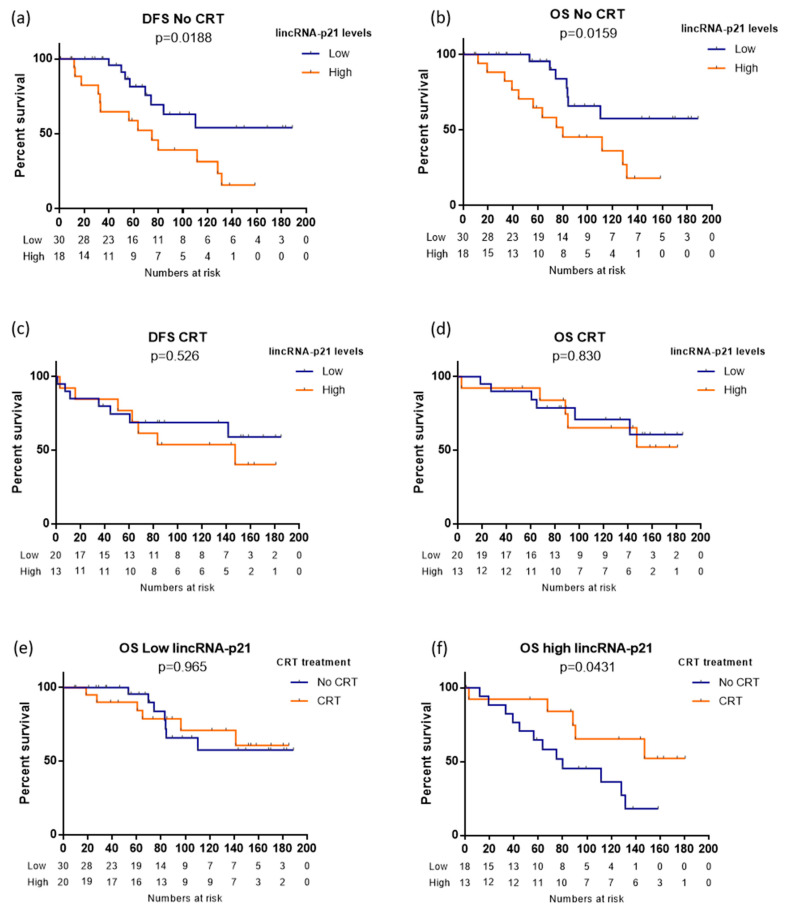
Disease-free survival (DFS) and overall survival (OS) according to lincRNA-p21 expression levels in (**a**,**b**) 48 rectal cancer patients not receiving radiotherapy and (**c**,**d**) 33 rectal cancer patients receiving radiotherapy. (**e**,**f**) Overall survival according to radiotherapy vs. no radiotherapy in (**e**) 50 rectal cancer patients with low levels of lincRNA-p21 and (**f**) 31 rectal cancer patients with high levels of lincRNA-p21. Log-rank test were used to calculate whether significant differences in survival times between groups were achieved.

Among RC patients not receiving adjuvant CRT, those with high levels of lincRNA-p21 had shorter DFS (NR versus 74.9 months; log-rank *p* = 0.018; Figure 3a) and OS (NR versus 80.1 months; log-rank *p* = 0.016; Figure 3b). However, this prognostic effect was lost among patients receiving adjuvant CRT (Figure 3c,d). Interestingly, when we analyzed the association between CRT and DFS and OS separately in the subgroups of patients with low or high lincRNA-p21, we observed different behaviors. Among patients with low lincRNA-p21 levels, there was no significant difference in OS between those receiving and not receiving CRT (log-rank *p* = 0.965; Figure 3e), while in those with high lincRNA-p21 levels, OS was longer in those receiving CRT (NR versus 80.1 months, log-rank *p* = 0.043; Figure 3f). Interestingly, in those patients treated with CT alone, without radiotherapy, OS was more similar to that in patients not receiving any adjuvant treatment (Supplementary Figure S1).

### 3.6. Exploratory Analysis of LincRNA-p21 Expression in Plasma Samples

**Figure 4:**
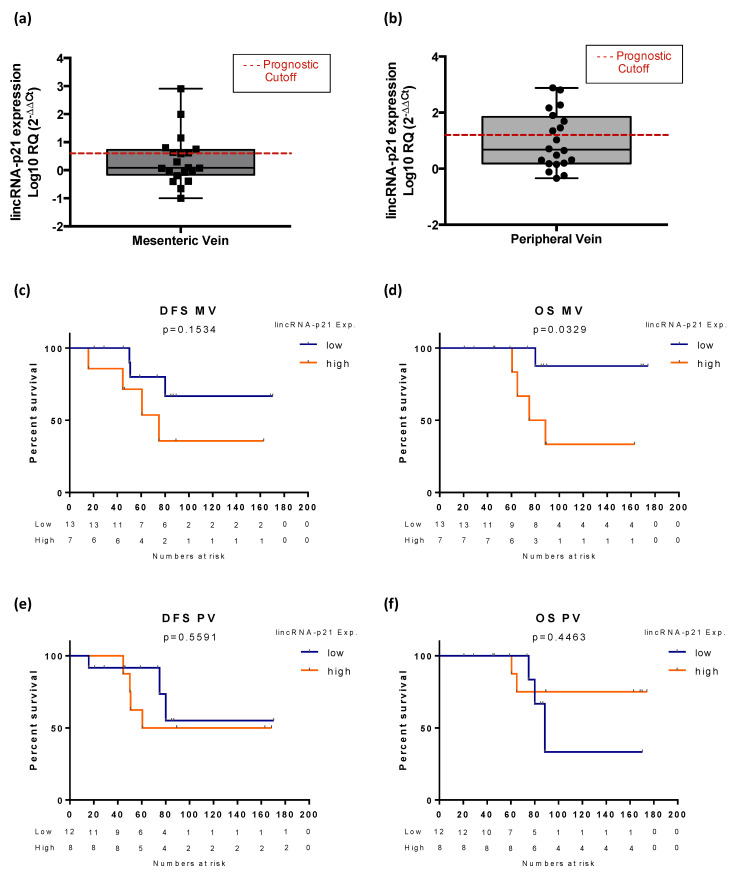
Expression levels of lincRNA-p21 in plasma obtained from (**a**) mesenteric vein and (**b**) peripheric vein (relative to normal colorectal tissue). The most optimal cutoff identified using x-Tile software and used for the prognostic analysis is indicated with a dashed red line. Disease-free survival (DFS) and overall survival (OS) according to lincRNA-p21 expression levels in plasma derived from (**c**,**d**) the mesenteric vein and (**e**,**f**) the peripheral vein in 20 rectal cancer patients. The log-rank test was used to calculate whether significant differences in survival times between groups were achieved.

Since lincRNA-p21 showed a major impact in RC patients, we decided to focus the analysis of plasmatic–lincRNA-p21 in this subgroup. Among the 20 RC patients with available blood samples (MV and paired PV), those with high plasmatic–lincRNA-p21 expression in plasma obtained from MV (Figure 4a) showed a trend toward shorter DFS (NR versus 74.90 months, *p* = 0.153; Figure 4c) and significantly shorter OS (NR versus 81.69 months, *p* = 0.032; Figure 4d). In contrast, there was no association between plasmatic–lincRNA-p21 expression (Figure 4b) and DFS (*p* = 0.5591) or OS (*p* = 0.4463) in samples from the PV (Figure 4e,f).

## 4. Discussion

In the present study, we have studied the clinical utility of lincRNA-p21 in CRC. The analysis of 177 CRC patients showed that despite lincRNA-p21 being downregulated in tumor tissue compared to normal tissue, its higher levels in tumor tissue positively correlated with advanced disease characteristics, including lymph node involvement and an advanced stage, and related to worse outcome. Moreover, we observed that lincRNA-p21 expression levels were associated with tumor location where RC patients had the highest lincRNA-p21 levels. In relation with this, we observed that the major prognostic impact was achieved in RC patients, whereas it was not significantly achieved in CC patients.

Several studies have explored the function and clinical role of lincRNA-p21 in CRC. An early study [22] including 66 CRC patients also reported that lincRNA-p21 was downregulated in tumor tissue compared to adjacent normal tissue and that high lincRNA-p21 expression correlated with factors associated with poor prognosis, such as higher TNM grade and vascular invasion. Moreover, they also showed that higher levels of lincRNA-p21 were observed in RC. However, no association with neither OS nor metastasis-free survival were found, maybe due to the reduced number of patients. However, in a more recent study [24] in 66 CRC patients, no significant differences were observed between tumor and adjacent normal tissue and lincRNA-p21 levels, but they found using ROC curve analysis that lincRNA-p21 could have an impact as diagnostic marker (AUC tumor versus normal tissue of 0.796). The prognosis impact was not studied in that work. Since lincRNA-p21 have been described as key element in the TP53 pathway, both papers studied the association with TP53 in patient samples, analyzing the correlation with TP53 mutational status [22] or the correlation with p53 mRNA levels [24]. However, no significant correlations with TP53 were observed in both papers.

Although no correlation with survival was observed in previous CRC studies, which was probably due to the small size of the cohorts analyzed, the prognosis impact of lincRNA-p21 has been confirmed in other tumors. In line with our results, high lincRNA-p21 levels were correlated with worse outcome in non-small-cell lung cancer [25] and prostate cancer [26], while in hepatocellular carcinoma [18] and diffuse large B lymphoma [27], high lincRNA-p21 expression has been related to better prognosis.

Since previous in vitro findings have linked lincRNA-p21 expression to radiation response [21], we explored this potential association in our cohort, where 41% of RC patients (33 of 81) received post-operative CRT. Interestingly, we found that high lincRNA-p21 patients benefit from CRT. We observed that the prognostic impact of lincRNA-p21 expression was lost in patients treated with CRT. Moreover, the subanalysis of patients according high or low lincRNA-p21 levels showed that among high lincRNA-p21 patients, those treated with CRT had longer OS than those who were not, while those with low lincRNA-p21 levels seemed to derive no survival benefit from CRT. No significant differences were observed in the group of patients treated only with CT, which indicates that lincRNA-p21 is related to radiotherapy response. These results are in line with previous in vitro studies showing that high lincRNA-p21 levels promoted apoptosis in CRC cell lines after radiation exposure by targeting the Wnt/β-Catenin pathway [21]. Similar results have also been reported in glioma cell lines, where a relationship between miR-146-5p and lincRNA-p21 correlated with sensitization to radiation therapy [28]. Taken together with these previous findings, our results indicate that lincRNA-p21 may be a promising predictive biomarker of radiotherapy benefit.

Finally, we performed an exploratory analysis to evaluate the potential utility of lincRNA-p21 as liquid biopsy biomarker. For this, we analyzed lincRNA-p21 levels in plasma obtained from two different sources: tumor-draining vein and PV. Our group have demonstrated previously the benefit of analyzing tumor-draining vein samples in front of PV in CCR [29,30] and also in lung cancer [31]. Our analysis was focused on RC, since in that group, we observed the major clinical impact. In MV but not in PV, we observed that high lincRNA-p21 levels were correlated with shorter OS. Although the number of samples studied was low, this exploratory analysis denotes a promising role of lincRNA-p21 as a liquid biopsy biomarker for resected RC patients that deserves to be further studied in larger cohorts. Moreover, several studies have previously pointed out the suitability of lncRNAs as a new source of biomarker in the field of liquid biopsy [32,33].

To date, the role of lincRNA-p21 in CRC has been studied in small patient cohorts or in vitro, and the present study is the first to explore its impact in a relatively large number of patients pointing out their relevance as prognostic biomarker, especially in RC patients. 

In summary, our findings indicate that lincRNA-p21 may be a useful prognostic biomarker in CRC, especially in RC where it also could play a role as an indicator of survival benefit from radiotherapy. Of note, these findings will benefit from validation in an independent cohort and from further studies to elucidate their mechanism of action, since validating the role of lincRNA-p21 as a prognostic marker could lead to the development of novel therapeutic strategies in CRC.

## 5. Conclusions

LincRNA-p21 is a marker of advanced disease and worse outcome in CRC with a major impact in RC patients, where it is clearly overexpressed. Moreover, lincRNA-p21 acts as a marker of radiotherapy response, since RC patients with high lincRNA-p21 levels benefit from post-operative CRT. Finally, the analysis of lincRNA-p21 levels in plasma from tumor-draining vein is a prognostic biomarker for resected RC patients.

## Figures and Tables

**Table 1:** Patient characteristics and association with disease-free survival (DFS) and overall survival (OS) according to the univariate analyses (Log Rank). Significant *p* values are shown in bold. CRC: colorectal cancer.

Characteristics	Value	All CRC Patients (*n* = 177)			Colon Cancer (*n* = 96)			Rectal Cancer (*n* = 81)		
		N (%)	DFS	OS	N (%)	DFS	OS	N (%)	DFS	OS
Sex	Male	103 (58.2)	0.644	0.311	52 (54.2)	0.473	0.928	51 (63)	0.081	0.111
	Female	74 (41.8)			44 (45.8)			30 (37)		
Age (years)	≤65	59 (33.3)	**0.002**	**0.001**	31 (32.3)	**0.02**	**0.012**	28 (34.6)	**0.024**	**0.002**
	>65	118 (66.7)			65 (67.7)			53 (65.4)		
ECOG PS *	0	8 (4.5)	**0.041**	**0.017**	6 (6.3)	**0.017**	**0.013**	2 (2.5)	0.405	0.292
	1	148 (84.2)			79 (82.3)			70 (86.4)		
	2	20 (11.3)			11 (11.5)			9 (11.1)		
T	1	11 (6.2)	0.467	0.266	3 (3.1)	0.373	0.446	8 (9.9)	0.761	0.391
	2	36 (20.3)			10 (10.4)			26 (32.1)		
	3	112 (63.3)			68 (70.8)			44 (54.3)		
	4	18 (10.2)			15 (15.6)			3 (3.7)		
N	0	120 (67.8)	0.233	0.418	63 (65.6)	0.385	0.344	57 (70.4)	0.659	0.858
	1	40 (22.6)			25 (26)			15 (18.5)		
	2	17 (9.6)			8 (8.3)			9 (11.1)		
Stage	I	37 (20.9)	0.314	0.554	10 (10.4)	0.258	0.353	27 (33.4)	0.763	0.994
	II	83 (46.9)			53 (55.2)			30 (37)		
	III	57 (32.2)			33 (34.4)			24 (29.6)		
Previous polyp	Yes	40 (22.6)	0.311	0.179	26 (27.1)	0.142	**0.03**	14 (17.3)	0.915	0.896
	No	137 (77.4)			70 (72.9)			67 (82.7)		
Mucin secretion	Yes	33 (18.6)	0.534	0.129	27 (28.1)	0.691	0.292	75 (92.6)	0.152	**0.054**
	No	144 (81.4)			69 (71.9)			6 (7.4)		
Relapse	Yes	33 (18.6)	---	---	17 (17.7)	---	---	16 (19.8)	---	---
	No	144 (81.4)			79 (82.3)			65 (80.2)		

* ECOG PS: Eastern cooperative oncology group performance status.

**Table 2:** Cox multivariate analyses of disease-free survival (DFS) and overall survival (OS). Bold for *p* < 0.05. RC: rectal cancer.

		DFS		OS	
	Factor	HR (95% CI)	*p* Value	HR (95% CI)	*p* Value
**CRC patients**	Advanced age	1.043 (1.016–1.071)	**0.02**	1.075 (1.042–1.110)	***p* < 0.001**
	Stage III	1.379 (0.687–2.769)	0.367	1.435 (0.676–3.046)	0.348
	No adjuvant treatment	0.778 (0.339–1.785)	0.554	0.658 (0.258–1.678)	0.380
	ECOG PS (0)	0.274 (0.056–1.341)	0.110	0.408 (0.081–2.046)	0.276
	High lincRNA-p21	1.747 (1.074–2.841)	**0.025**	1.884 (1.104–3.217)	**0.020**
**RC Patients**	Advanced age	1.032 (0.996–1.069)	0.087	1.064 (1.017–1.113)	**0.007**
	Male	0.570 (0.290–1.119)	0.102	-	-
	Mucin secretion	-	-	2.468 (0.833–7.317)	0.103
	Stage III	0.769 (0.148–4.013)	0.756	0.606 (0.095–3.861)	0.596
	No adjuvant treatment	0.924 (0.409–2.090)	0.850	0.996 (0.423–2.345)	0.992
	High lincRNA-p21	1.986 (1.013–3.895)	**0.046**	1.318(0.562–3.091)	0.562

## Supplementary Materials

The following are available online at https://www.mdpi.com/2075-1729/10/9/172/s1, Figure S1: OS of patients according adjuvant treatment strategy administered (No treatment, CT or CRT) in patients with high (a) high lincRNA-p21 or (b) low lincRNA-p21 levels.

## Abbreviations

CC	Colon cancer
CI	Confidence intervals
CRC	Colorectal cancer
CT	Chemotherapy
CTR	Chemoradiotherapy
DFS	Disease-free survival
HR	Hazard ratios
MDPI	Multidisciplinary Digital Publishing Institute
MV	Mesenteric vein
NT	Normal tissue
OS	Overall survival
PV	Peripheral vein
RC	Rectal cancer
TT	Tumor tissue
