# Supplementary figures and images for: LincRNA-p21 Levels Relates to Survival and Post-Operative Radiotherapy Benefit in Rectal Cancer Patients
Source: Life (Basel). 2020 Aug 31;10(9):172. doi: 10.3390/life10090172 (PMC7555220; doi:10.3390/life10090172)

Supplementary Figure 1

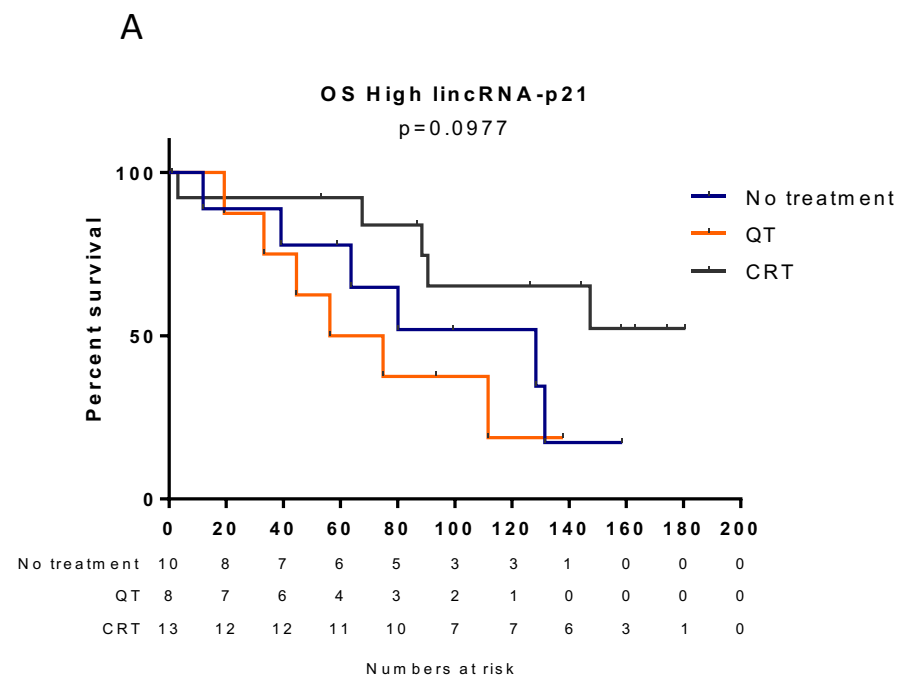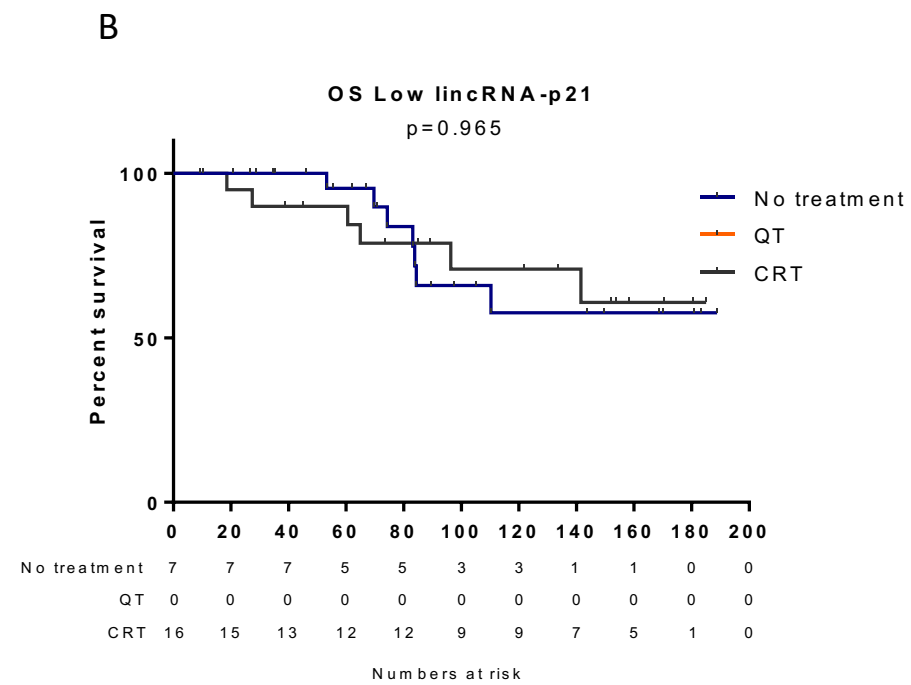

Supplement: Supplementary file 1 [file life-10-00172-s001.pdf]
